# Deep Q-networks with web-based survey data for simulating lung cancer intervention prediction and assessment in the elderly: a quantitative study

**DOI:** 10.1186/s12911-021-01695-4

**Published:** 2022-01-04

**Authors:** Songjing Chen, Sizhu Wu

**Affiliations:** Institute of Medical Information, Chinese Academy of Medical Sciences/Peking Union Medical College, Beijing, China

**Keywords:** Lung cancer, Deep Q-networks, Early intervention, Aged, Primary prevention

## Abstract

**Background:**

Lung cancer screening and intervention might be important to help detect lung cancer early and reduce the mortality, but little was known about lung cancer intervention strategy associated with intervention effect for preventing lung cancer. We employed Deep Q-Networks (DQN) to respond to this gap. The aim was to quantitatively predict lung cancer optimal intervention strategy and assess intervention effect in aged 65 years and older (the elderly).

**Methods:**

We screened lung cancer high risk with web-based survey data and conducted simulative intervention. DQN models were developed to predict optimal intervention strategies to prevent lung cancer in elderly men and elderly women separately. We assessed the intervention effects to evaluate the optimal intervention strategy.

**Results:**

Proposed DQN models quantitatively predicted and assessed lung cancer intervention. DQN models performed well in five stratified groups (elderly men, elderly women, men, women and the whole population). Stopping smoking and extending quitting smoking time were optimal intervention strategies in elderly men. Extending quitting time and reducing smoked cigarettes number were optimal intervention strategies in elderly women. In elderly men and women, the maximal reductions of lung cancer incidence were 31.81% and 24.62% separately. Lung cancer incidence trend was deduced from the year of 1984 to 2050, which predicted that the difference of lung cancer incidence between elderly men and women might be significantly decreased after thirty years quitting time.

**Conclusions:**

We quantitatively predicted optimal intervention strategy and assessed lung cancer intervention effect in the elderly through DQN models. Those might improve intervention effects and reasonably prevent lung cancer.

## Introduction

The morbidity and mortality of lung cancer in many countries have increased significantly in past decades [1]. Lung cancer incidence has accounted for 11.6% of cancer new cases in 2018 according to World Cancer Report 2020 [1], which has a higher incidence in the elderly (aged 65 years and older). Elderly population is growing rapidly in recent years. One in six people in the world will be over age 65 (16%) by 2050, which is up from one in eleven in 2019 (9%) [2]. Lung cancer intervention measures [3–5] are effective ways of reducing lung cancer incidence.

Many researches have been actively carried out to help detect lung cancer early, e.g. lung cancer screening [6–8], and reduce lung cancer mortality, e.g. intervention for people with lung cancer [9–11]. The Lung, Prostate, Colorectal, and Ovarian (PLCO) cancer screening trial used annual screening with chest radiograph to evaluate the effect on mortality for lung cancer screening [6]. The National Lung Screening Trial (NLST) was conducted to screen the usage of low-dose computed tomography (LDCT) with reducing mortality of lung cancer [7]. Zahnd et al. analyzed the utilization of computed tomography (CT) in lung cancer screening through Behavioral Risk Factor Surveillance System (BRFSS) [8]. Manuel and colleagues assessed the feasibility of a multimodal physical activity, nutrition and palliative symptom management intervention in advanced lung cancer [9]. Lung cancer screening and intervention might be important to help detect lung cancer early and reduce the mortality, but little was known about lung cancer intervention strategy associated with intervention effect for preventing lung cancer. We attempted to simulate lung cancer intervention process in the elderly to predict the optimal intervention strategy and assess the intervention effect, which might improve intervention efficiency and provide evidence for precise intervention.

Reinforcement learning is a branch of machine learning, which emphasizes the action based on its environment to obtain the maximum expected effect. Reinforcement learning uses a learning method through iteratively updating. When receiving input samples, reinforcement learning uses the current model to guide next action, updates the model after getting a reward form next action, and iteratively repeats until the model converging. Reinforcement learning is regularly applied to realize optimal problem-solving strategies. Deep learning is used to simulate the multi-level information processing approach of human brain to extract representative characteristics. Deep reinforcement learning [12, 13] combines the decision-making ability of reinforcement learning with the perception ability of deep learning. Deep Q-Networks (DQN) [14] is a representative method of deep reinforcement learning, which can achieve hierarchical representation of input information and simulate intervention process with high accuracy that can be effectively used for lung cancer detection [15–17]. But DQN has been rarely used for predicting optimal intervention strategy to prevent lung cancer so far. Issa and colleagues used deep reinforcement learning model for early detection of lung nodules in thoracic CT images [15]. Tseng et al. demonstrated that the radiation detection in radiotherapy for lung cancer patients based on deep reinforcement learning [17]. However, some deep learning methods have been applied to improve the intervention strategy to treat lung cancer [18, 19]. Rongfang and colleagues adopted multi-objective ensemble deep learning to predict high risk of treatment failure after radiotherapy in lung cancer patients [18]. Comparing with other deep learning methods [18–20], DQN employs convolution neural network (CNN) to approximate objective function and build experience replay in the model training process, which could improve accuracy and efficiency in model training process. Therefore, this study was an exploration of high-performance DQN, which was developed to predict intervention strategy and assess intervention effect to prevent lung cancer.

The purposes of this study were to: (i) quantitatively predict optimal intervention strategy; and (ii) assess lung cancer intervention effect in the elderly through DQN modelling. We described the development of DQN models and conducted intervention simulation based on our previous identified lung cancer risk factors in the elderly [21], which mainly targeted non-small cell lung cancer. In our previous study [21], we had identified high risk factors in elderly men, e.g. smoking frequency, time since quitting and use of e-cigarettes, and risk factors in elderly women, e.g. time since quitting, smoked at least 100 cigarettes and smoking frequency. These risk factors were employed to screen lung cancer high risk of elderly in this study. We deduced the lung cancer optimal intervention strategy for elderly people and quantitatively simulated the lung cancer incidence trend, which could help the public raise awareness of lung cancer intervention and prevent lung cancer efficiently.

## Material and methods

### Data collection and preparation

**Fig. 1:**
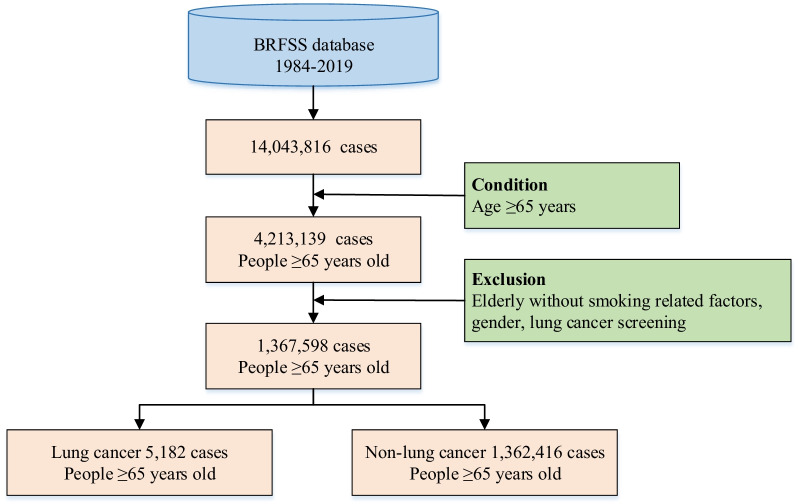
The flowchart of data selection

The health-related surveys data from BRFSS [22] were used in this study. BRFSS collected United States residents’ data on health risk behaviors and chronic health conditions [22], which involved various risk factors of lung cancer and its prevalence situation, such as age, body mass index, smoking frequency, smoking start age, smoking intensity, time since quitting smoking, personal cancer history, family history of cancer, e-cigarette use, asthma history, chronic obstructive pulmonary disease (COPD) history, et al. The data selection flowchart was shown in Fig. 1. The whole population (14,043,816 cases) of the survey were aged older than 18 years old. Of those, 47.39% (6,655,364 cases) were men and 52.61% (7,388,452 cases) were women. By leveraging data preprocessing, some cases which had missing values were excluded, e.g. missing smoking related factors, gender, lung cancer screening. The elderly population were those aged 65 years old and older according to international age threshold for the elderly in the developed countries. 1,367,598 elderly cases were obtained totally. The proportion of men 65 years and older was 48.36% (661,370 cases). In order to analyze the specificity of the characteristics of lung cancer incidence in the elderly, men aged 18 years and older and women aged 18 years and older, as well as the whole population, were included in the study to compare with the elderly. In all, five stratified groups: men aged 65 years and older (elderly men), women aged 65 years and older (elderly women), men aged 18 years and older (men), women aged 18 years and older (women) and the whole population (all), were obtained in this study.

We also selected environmental data from US Environmental Protection Agency (EPA) [23] website, which related to particulate matter (PM), carbon monoxide (CO), lead (Pb), Ozone, sulfur dioxide (SO_2_), nitrogen dioxide (NO_2_), 24-h average temperature, relative humidity, wind speed, duration of sunshine, precipitation, atmospheric pressure and indoor radon. The Environmental data were linked to BRFSS through the collection date, which could integrate these two datasets together.

### Data analysis

**Fig. 2:**
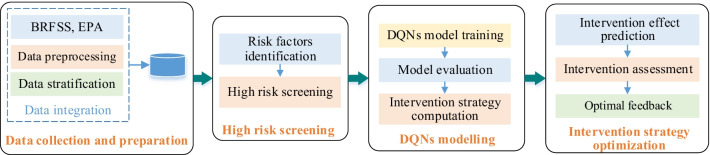
Workflow of lung cancer intervention prediction and assessment

We adopted DQN model to predict lung cancer intervention strategy and assess intervention effect for lung cancer high risk. The workflow of this study was shown in Fig. 2. Firstly, we separately screened lung cancer high risk in five stratified groups. Secondly, DQN models were developed to deduce lung cancer intervention strategy in different stratifications. Thirdly, lung cancer incidences were computed according to corresponding intervention strategy, and intervention effects were deduced through DQN models. Lastly, we assessed lung cancer intervention effect to derive optimal intervention strategy.

#### Lung cancer high risk screening

Timely high risk screening and early intervention [24] might reduce the incidence of lung cancer. We screened risk factors for lung cancer of elderly men and women through our previous study [21]. In elderly men, smoking frequency and time since quitting (i.e. how long has it been since the respondent last smoked a cigarette) were the top two risk factors for lung cancer [21]. Thus, according to the risk factors, the lung cancer high risk of elderly men was screened. Time since quitting and smoked at least 100 cigarettes (i.e. smoked at least 100 cigarettes in respondent’s entire life) were the high risk factors in elderly women [21]. Similarly, we screened lung cancer high risk of elderly women. We obtained 103,629 high risk elderly people and developed intervention simulation to predict lung cancer optimal intervention strategy in elderly men and women.

#### Deep Q-networks modelling

DQN was a value-based reinforcement learning algorithm, which used CNN to approximate value functions. DQN models’ inputs were risk factors of high risk people, which were obtained from our previous study [21], e.g. smoking frequency, cancer history, asthma history, radiation, use of e-cigarette, time since quitting, physical activity. And models’ outputs were optimal intervention strategies which were deduced from target value functions. Value functions were trained using CNN to get close to maximal intervention effect as much as possible.

We adopted Q-learning method to develop networks and computed the loss function. The loss function was shown in Eq. (1). *Q* was output value function of neural network, which represented maximum cumulative intervention effect of intervention strategy *a* from risk state *s*; *Q(s, a; θ*_*i*_*)* was output of current network; *Q*_*i*_ was output of the target network; *θ* was mean squared error of network parameters; and *ρ(s, a)* was probability distribution of risk state *s* and intervention strategy *a*.

1 $$L_{i} (\theta_{i} ) = E_{s,a\sim \rho ( \cdot )} [(Q_{i} - Q(s,a;\theta_{i} ))^{2} ]$$

We iteratively updated weights of optimization loss function using the stochastic gradient descent (SGD) function, as shown in Eq. (2). *Q(s′, a′; θ*_*i-1*_*)* was the target network output; *Q(s, a; θ*_*i*_*)* was current network output; *r* was intervention effect of current network; *ε* was intervention environment; and *γ* was discount factor and between 0 and 1.

2 $$\nabla_{{\theta_{i} }} L_{i} (\theta_{i} ) =\, E_{s,a\sim \rho ( \cdot );s^{\prime}\sim \varepsilon } [(r + \gamma \mathop {\max }\limits_{a^{\prime}} Q(s^{\prime},a^{\prime};\theta_{i - 1} ) - Q(s,a;\theta_{i} ))\nabla_{{\theta_{i} }} Q(s,a;\theta_{i} )]$$

Then, by leveraging SGD function, the current value function was getting close to target value function as much as possible. Output target value function *Q*_*i*_ was combined with optimal intervention strategy *a* and risk state *s*, which was in Eq. (3) and could be used to deduce optimal intervention strategy.

3 $$Q_{i} = E_{s^{\prime}\sim \varepsilon } [r + \gamma \mathop {\max }\limits_{a^{\prime}} Q(s^{\prime},a^{\prime};\theta_{i - 1} )|s,a]$$

Rectified linear unit was activation function in this study, which was integrated into convolutional layer. The model consisted of one input layer, three convolutional layers, one fully connected layer and one output layer. We adopted input neurons 32 × 32, convolution kernels 5 × 5, 4 × 4 and 3 × 3 of three convolutional layers respectively and four output neurons. Ten-fold cross-validation was used to evaluate the model, which randomly divided the dataset into ten parts and took turns using nine parts for model training and one part for model testing. Python script and PyTorch framework were employed in Ubuntu programming environment based on Docker platform for model training in this study. We separately trained five DQN models of elderly men, elderly women, men, women and the whole population. Intervention strategies of these five groups were derived from their DQN models.

#### Intervention strategy optimization

**Fig. 3:**
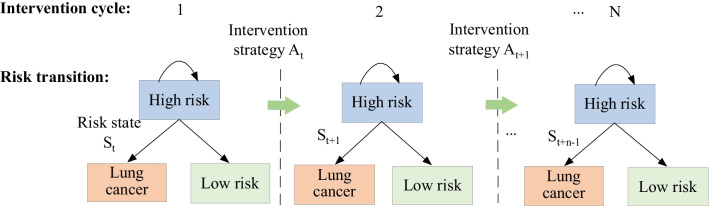
Diagram of risk state transitions of high risk for lung cancer occurrence

(i) Intervention effect prediction
  The high risk was a risk state of lung cancer occurrence in this study. There were other risk states as well, such as low risk and lung cancer. Once intervention strategy was conducted, risk state might change, which was risk state transition. Risk state transitions of high risk included from high risk to low risk, from high risk to lung cancer, from high risk to high risk. We used the probability of risk state transition to assess the intervention effect of intervention strategy in this study. Similar intervention effect predictions in different stratifications were developed.
(ii) Lung cancer intervention assessment
  Probabilities of risk state transitions were assessed in different groups. As in Fig. 3, we described risk state transitions of high risk in multiple intervention cycles, where S_t_ was the set of risk states at time t; A_t_ was the set of intervention strategies at time t. We computed probabilities of risk state transition of high risk in different intervention cycles. We comprehensively assessed the intervention effects in elderly men and women using lung cancer incidence.
(iii) Optimal feedback

Based on intervention effect assessment, we employed the reduction of lung cancer incidence to reflect the effectiveness of intervention strategy. The intervention strategy could bring the largest reduction of lung cancer incidence than other strategies, which would be considered as the optimal intervention strategy. Otherwise, this intervention strategy would be adjusted using feedback mechanism. The whole process was reworked as shown in Fig. 2 and intervention effect was comprehensively evaluated until optimal intervention strategy was deduced.

### Model performance evaluation

To evaluate the models, we adopted ten-fold cross-validation. Accuracies and area under the receiver operating characteristic curve (AUROC) of five models were computed separately. Then we compared DQN models with support vector machines (SVM), random forest and multiple logistic regression in five groups to conduct method comparison.

## Results

### Lung cancer intervention effects

Lung cancer intervention effects of stratified elderly were derived from DQN models and listed in Table 1, which showed top five effective intervention scenarios. In Table 1(a), the maximal reduction of lung cancer incidence in elderly men was given Scenario 1, that was the intervention strategy in Scenario 1 was the optimal intervention strategy of elderly men. Similarly, the intervention strategy in Scenario 1 in Table 1(b) was the optimal intervention strategy of elderly women.

**Table 1:** Lung cancer intervention effect of the elderly

Scenario	Intervention strategy	Lung cancer incidence (per 100,000)	Incidence reduction (%)	Odds ratio
*(a) Lung cancer intervention effect of elderly men*				
1	From everyday smoking to quitting; quit smoking from within 1 month to 5 years or more	248.94	31.81	1.103
2	From someday smoking to quitting; quit smoking from within 1 month to 5 years or more	275.25	24.14	1.217
3	From everyday smoking to someday smoking; quit smoking from within 1 month to 1–5 years or more	303.60	16.65	1.178
4	Quit smoking from within 1 month to 5 years or more; from use e-cigarette to quit e-cigarette	323.53	11.14	1.358
5	Quit smoking from 1–3 months to 10 years or more; from smoked at least 100 cigarettes to quitting	334.78	8.04	1.026
*(b) Lung cancer intervention effect of elderly women*				
1	Quit smoking from within 1 month to 5 years or more; from smoked at least 100 cigarettes to quitting	183.73	24.62	1.214
2	Quit smoking from within 1 month to 5 years or more; from everyday smoking to quitting	195.39	19.77	1.031
3	Quit smoking from 1–3 months to 5 years or more; from someday smoking to quitting	206.68	15.06	1.095
4	Quit smoking from within 1 month to 10 years or more; from smoked at least 100 cigarettes to quitting	217.50	10.53	1.274
5	Quit smoking from 6 months to 1 year to 10 years or more; from smoked at least 100 cigarettes to quitting	224.79	7.74	1.310

In Scenario 1 of Table 1(a), when elderly men reduced smoking frequency from everyday smoking to quitting and extended quitting smoking time to more than 5 years, lung cancer incidence decreased from 365.07 per 100,000 to 248.94 per 100,000, which brought the maximal reduction of 31.81% in elderly men. Therefore, intervention strategy of Scenario 1 was the most effective intervention combination pattern in elderly men among the top five scenarios in Table 1(a). However, intervention strategies which listed in Scenarios 2–5, could effectively reduce the lung cancer incidence, Scenario 1 showed the optimal intervention strategy in elderly men.

Extending the time since quitting smoking and reducing smoked cigarettes number, as shown in Scenario 1 of Table 1(b), were the optimal intervention strategy, which was more effective than other strategies in elderly women. It brought the maximal reduction of 24.62% for lung cancer incidence from 243.74 per 100,000 to 183.73 per 100,000. Comparing the maximal reduction of lung cancer incidence in elderly men and women, i.e., 31.81% and 24.62%, we found that conducting interventions in elderly men were more effective than elderly women.

### Optimal intervention strategies

**Fig. 4:**
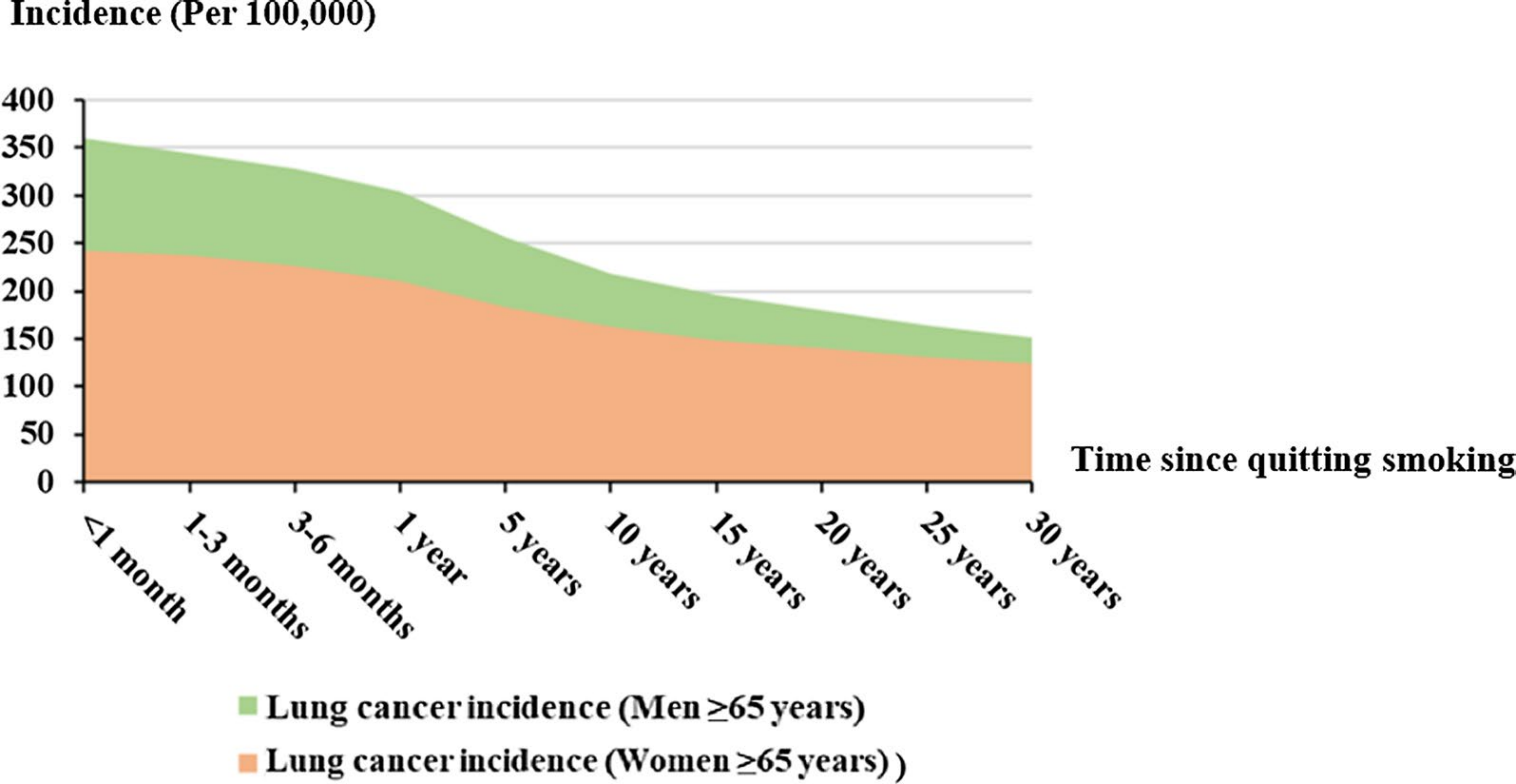
Relationship between incidence and time since quitting smoking in the elderly

The optimal intervention strategy in elderly men were quitting smoking and extending the quitting smoking time more than 5 years. Meanwhile, quitting smoking more than 5 years and reducing smoking cigarettes number were the optimal strategy in elderly women. Based on optimal intervention strategies, the relationship between lung cancer incidence and time since quitting smoking of elderly men and women was shown in Fig. 4.

Lung cancer incidences of elderly men and women were decreased with quitting time extended. Moreover, the disparity of lung cancer incidence between elderly men and women would decrease in thirty years quitting smoking time.

### Lung cancer incidence trends

**Fig. 5:**
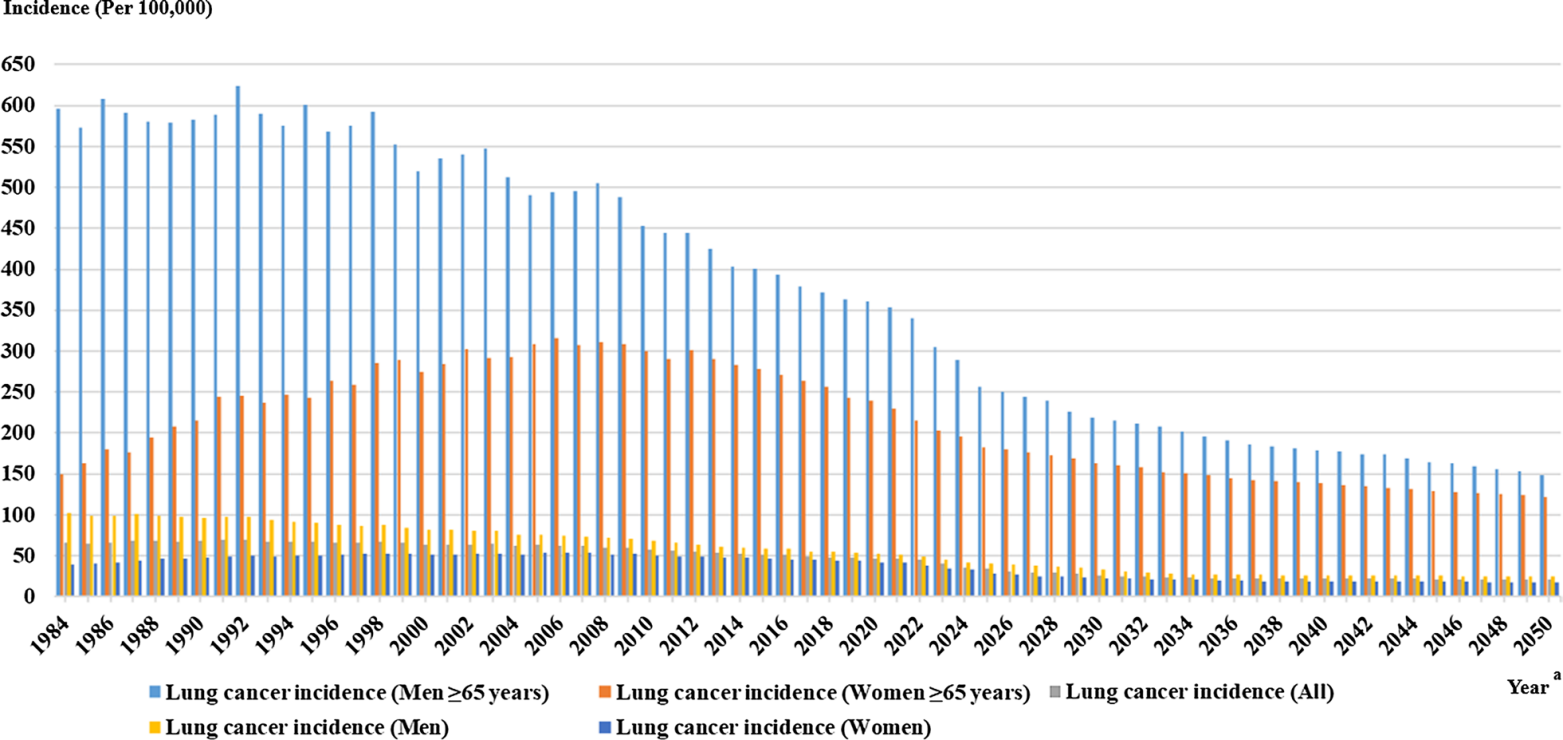
Lung cancer incidence trends of stratified population

We computed lung cancer incidence of elderly men, elderly women, men, women and the whole population, from the year of 1984 to 2019, and predicted their lung cancer incidences through DQN models during 2020–2050, as shown in Fig. 5. We quantitatively analyzed lung cancer incidence trends while using optimal intervention strategies. During 1984–2050, lung cancer incidence of elderly men reduced fast, which decreased about 76.08% from 623.41 to 149.13 per 100,000. In elderly women, lung cancer incidence decreased 61.32% from 316.25 to 122.32 per 100,000 during 1984–2050. Lung cancer incidences in the whole population, men and women were all decreased, but elderly men and elderly women had much more significant reduction of lung cancer incidence than them.

### Deep Q-networks models performances

DQN models performances were illustrated in Table 2. Accuracies and AUROC of DQN models performed well. We employed the model of the whole population as baseline to demonstrate models’ performances. Accuracies of elderly men, elderly women, men and women were 93.8%, 94.8%, 90.6% and 92.6% were high than 89.5% for the whole group. In addition, AUROCs in other four groups were higher than in the whole group. Therefore, proposed DQN models for elderly people with high performance were reliable and efficient.

**Table 2:** Performances of DQN models

Model	Accuracy	AUROC ^a^	*P* value ^b^
Men ≥ 65 years	0.938	0.903	.003
Women ≥ 65 years	0.948	0.915	.002
Men	0.906	0.924	.015
Women	0.926	0.921	.002
All people	0.895	0.893	.002

^a^*AUROC* area under the receiver operating characteristic curve

^b^*P* value: *P* < .05 was considered to indicate statistical significance

### Comparison with other methods

We compared with SVM, random forest and multiple logistic regression to evaluate the effectiveness of DQN method, which were generally employed to predict the lung cancer intervention. In elderly men, stopping smoking and extending the time since quitting were the optimal intervention strategy through SVM, random forest and multiple logistic regression models, which were consistent with DQN model. But the accuracies of them were separately lower 8.5%, 9.2% and 9.5% than DQN model as shown in Table 2 and Table 3. AUROC value was also lower than DQN model. And training speed was slower than DQN model. Similarly, we obtained models for other four groups using the three methods as shown in Table 3, which performed slightly worse than DQN models. Therefore, DQN method was more effective than several supervised learning and statistical methods, such as SVM, random forest and multiple logistic regression.

**Table 3:** Performances of other models

Model	SVM			Random forest			Multiple logistic regression		
Index	Accuracy	AUROC^a^	*P* value^b^	Accuracy	AUROC	*P* value	Accuracy	AUROC	*P* value
Men ≥ 65 years	0.853	0.822	.003	0.846	0.837	.002	0.843	0.805	.002
Women ≥ 65 years	0.838	0.835	.015	0.826	0.819	.001	0.826	0.796	.003
Men	0.841	0.813	.001	0.817	0.793	.003	0.833	0.822	.015
Women	0.812	0.786	.002	0.801	0.786	.015	0.796	0.815	.003
All people	0.803	0.794	.015	0.787	0.813	.002	0.807	0.799	.015

^a^*AUROC* area under the receiver operating characteristic curve

^b^*P* value: *P* < .05 was considered to indicate statistical significance

## Discussion

### Principal findings

We derived optimal intervention strategies of stratified elderly and quantitatively assessed intervention effects of different strategies through DQN models, which could improve the efficiency of lung cancer prevention. We demonstrated the optimal intervention strategy for elderly men and women, by which the maximal reductions of lung cancer incidence were 31.81% and 24.62% separately. Lung cancer incidence trend was deduced from the year of 1984 to 2050, which predicted that the difference of lung cancer incidence between elderly men and women might be significantly decreased after thirty years quitting time. Proposed DQN models performed well in intervention strategy prediction and intervention effect assessment, which were more effective than SVM, random forest and multiple logistic regression. Accuracies of stratified groups using DQN models ranged from 89.5 to 94.8% and AUROCs ranged from 0.893 to 0.924. DQN models were integrated by CNN of deep learning and Q-learning of reinforcement learning, which could perform high accuracy and save model training time. Moreover, DQN could obtain optimal intervention strategy from high dimensional input risk factors, which had high efficiency in strategy optimization comparing with other models [25–29].

Although previous researches of lung cancer intervention mainly focused on patients with diagnosed lung cancer [30, 31] or advanced lung cancer patients [9, 32], our study targeted to high risk elderly who had not developed lung cancer, which would be more conducive to prevent and control lung cancer. Junga investigated the exercise interventions in patients with lung cancer during chemotherapy regarding physiological and psychological outcomes [30]. Kureshi et al. suggested that support vector machines and decision trees were a promising approach for personalized therapeutic interventions in non-small cell lung cancer [32]. Deep reinforcement learning was tried to explore lung cancer optimal intervention strategy of high risk elderly due to its high-performance in solving optimal problems. The representative deep reinforcement learning models were built for lung cancer detection [14] and optimal treatment regimens discovery [33]. Liu et al. focused on the deep reinforcement learning for lung cancer detection and diagnosis, which could significantly improve the treatment effect and prolong survival [14]. These related studies supplied research feasibility and basis for optimal intervention strategy prediction and intervention outcome assessment through deep reinforcement learning. Meanwhile, conducting intervention prediction and assessment for lung cancer high risk in elderly might help reduce lung cancer incidence and financial burden of the nation and family.

The proposed results demonstrated DQN models predicted optimal intervention strategies, and assessed intervention effect in the stratified elderly. Stopping smoking and extending quitting smoking time were optimal intervention strategies in elderly men, which could reduce 31.81% of lung cancer incidence. Extending quitting time and reducing smoked cigarettes number were optimal intervention strategies in elderly women, which brought 24.62% reduction of lung cancer incidence. With the time increasing, lung cancer incidence in elderly men and women was gradually decreasing. After thirty years quitting time, the difference of lung cancer incidence between elderly men and women would become smaller. Jihyoun et al. revealed that the existing disparities in lung cancer by gender would disappear by the mid-2040s, and lung cancer rates would become roughly equal between men and women [34]. Gredner and colleagues suggested that cancer incidence could be reduced by implementing tobacco control policies in Germany [35]. Conducting intervention in the elderly had great effect on preventing lung cancer, especially in men aged 65 years and older. Therefore, effective interventions could decrease lung cancer incidence in the elderly and improve their life quality.

### Comparison with prior work

Previous researches developed multiple types of models [32, 36–40], which were used for lung cancer intervention prediction. We predicted optimal intervention strategies for lung cancer and assessed intervention effect from proposed models with high accuracy and AUROC. In terms of intervention strategy optimization, DQN model had great advantages in handling large-scale and high-performance in computation, which was more effective than several supervised learning, e.g., SVM, random forest, and statistical method, e. g., multiple logistic regression. DQN adopted prioritized experience replay [41], which could not only improve the intervention effect but accelerate model training speed. DQN model was developed with high efficiency and optimized performance.

### Limitations

Several limitations and assumptions existed in this study. We derived the optimal intervention strategy associated with its intervention effect based on DQN model that provided interpretable results, but clinical applicability of models should be evaluated in future study. Moreover, the study was conducted using web-based survey data, however there might be some new insights while survey data was combined with clinical data. Therefore, it was recommended to evaluate findings of this study in clinical environment. Additionally, we focused on the method feasibility for intervention prediction. Nevertheless, we assumed that the physical condition of elderly didn’t change much with age increasing, which would be improved in future work.

## Conclusion

DQN models were developed to quantitatively predict and assess lung cancer intervention in the elderly in this study. We demonstrated optimal intervention strategies for elderly men and women associated with their intervention effects. Lung cancer incidence trend was deduced and provided evidence that the difference of lung cancer incidence between elderly men and women might be significantly reduced after thirty years quitting time. This study could improve intervention effects and bring reasonable prevention of lung cancer. Proposed approach might explore a novel idea in cancer intervention prediction. It might be extended to other diseases to help physicians make decisions.

## Data Availability

The datasets generated or analysed during the current study are available from the corresponding author on reasonable request.

## Abbreviations

BRFSS: Behavioral risk factor surveillance system
DQN: Deep Q-networks
CNN: Convolution neural network
AUROC: Area under the receiver operating characteristic curve
LDCT: Low-dose computed tomography
CT: Computed tomography
